# Incidence of P200 pemphigoid: A nationwide study

**DOI:** 10.1111/jdv.70370

**Published:** 2026-03-07

**Authors:** Fabienne Jouen, Marion Carrette, Marie‐Laure Golinski, Maud Maho‐Vaillant, Alexis Lefebvre, André Gillibert, Pascal Joly, Vivien Hébert

**Affiliations:** ^1^ Department of Immunology and Biotherapy Rouen University Hospital and INSERM U 1234 Rouen France; ^2^ Department of Dermatology Rouen University Hospital and INSERM U 1234, Centre de référence des maladies bulleuses Autoimmunes, Normandie University Rouen France; ^3^ Department of Biostatistics Rouen University Hospital Rouen France

**Keywords:** anti‐p200 pemphigoid, autoimmune bullous diseases, cell‐based immunoassay, incidence, laminin β4

## Abstract

**Background:**

Anti‐p200 pemphigoid (P200) is a rare subepidermal autoimmune blistering disease (AIBD) related to anti‐laminin γ1 autoantibodies (Abs). It has been recently reported that laminin β4 was frequently recognized by sera from patients with P200.

**Objectives:**

To evaluate the incidence of P200 in France using a new recombinant anti‐laminin β4 serological assay.

**Methods:**

This multicentre retrospective study was conducted in the Immunology Laboratory of the French reference centre for AIBD from January 2018 to March 2023. Sera suspected of corresponding to P200 were tested using the new anti‐LAMB4 cell‐based immunoassay (CBIA) and/or immunoblotting (IB) on dermal extract. The incidence of P200 was then estimated based on its incidence rate relative to those of BP and pemphigus over the same period.

**Results:**

116 patients with sera labelling the dermal side of salt‐split skin by IIF and negative for other junctional AIBD were recorded. Among them, 23 patients' sera had already been tested positive for P200 by dermal IB. Thus, 93 patients' sera were tested with both the anti‐LAMB4 CBIA and IB: 85 were positive with the CBIA (91%) vs. 44 with IB (47%, *p* < 0.0001). Only two were positive with IB alone. Overall, 110 patients had anti‐p200 Abs. Subsequently, we compared the frequency of serological detection of P200 to that of BP and pemphigus based on data from centres that exhaustively submitted their sera to our reference laboratory. According to the incidence of BP and pemphigus in France (21.7 and 1.85 cases per million inhabitants per year, respectively), the incidence of P200 was estimated to range from 0.23 to 0.83 cases per million inhabitants per year. This corresponds to approximately 16–57 new cases annually in France, which is approximately 2‐ to 4‐fold more frequent than *epidermolysis bullosa acquisita*.

**Conclusions:**

This study shows that P200 may be much more frequent than initially estimated.

## Plain Language Summary

**Anti‐p200 pemphigoid incidence in France**.

Anti‐p200 pemphigoid is a rare autoimmune blistering disease involving the skin and mucous membranes. Since clinical features of anti‐p200 pemphigoid may be heterogeneous, the diagnosis relies on the detection of circulating anti‐p200 antibodies. Up to recently, the reference assay to identify anti‐p200 autoantibodies, restricted to specialized laboratories, was immunoblot analysis on dermal extract or on recombinant laminin γ1. A new anti‐laminin β4 cell‐based immunoassay (CBIA) has been recently developed for the detection of anti‐p200 antibodies.

To assess the sensitivity of this new assay, we tested all sera labelling the dermal side of salt‐split skin by indirect immunofluorescence (IIF) and compared the results with those provided by the immunoblot analysis on dermal extracts. Given the high sensitivity of the anti‐laminin β4 CBIA, which is in fact close to that of ELISA for the detection of anti‐desmoglein and anti‐BP180 in pemphigus and bullous pemphigoid (BP), respectively; we then estimated the incidence of anti‐p200 pemphigoid relative to that of pemphigus and BP.

One‐hundred‐and‐ten out of the 141 sera tested (71%) contained anti‐laminin β4 Abs when tested with the anti‐LAMB4 CBIA assay. Among the 93 sera analysed with both the anti‐LAMB4 CBIA and dermal immunoblotting, 85 were positive with the CBIA (91%) vs. 44 by immunoblotting (47%, *p* < 0.0001). According to the incidence of BP and pemphigus in France, the incidence of anti‐p200 pemphigoid was estimated at between 0.32 and 0.62 cases per million inhabitants per year, suggesting that anti‐p200 pemphigoid is much more frequent than initially reported.

## Graphical abstract

The anti‐LAMB4 cell‐based immunoassay is a new serological technique that is far superior to dermal immunoblotting for detecting autoantibodies directed against the P200 protein. This method allows a re‐evaluation of the incidence of P200 pemphigoid, which appears to be considerably more frequent than epidermolysis bullosa acquisita.

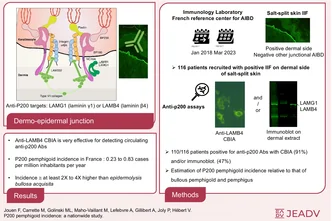

**Why was the study undertaken?**

- Anti‐p200 pemphigoid is a rare autoimmune blistering disease with autoantibodies directed against a 200 kDa molecule initially identified as laminin γ1.
- The disease incidence may be underestimated due to the limited availability and sensitivity of the current serological assay: immunoblot on dermal extract.
- A new sensitive cell‐based serological assay has been recently developed to detect anti‐p200 antibodies directed against laminin β4.

**What does this study add?**

- This new assay which detects autoantibodies directed against laminin β4 has a higher sensitivity than immunoblot on dermal extract.
- The very high proportion of sera containing anti‐laminin β4 antibodies among sera labelling the dermal side of salt‐split skin by indirect immunofluorescence suggests that anti‐p200 pemphigoid is more frequent than previously estimated, and in particular, much more frequent than epidermolysis bullosa acquisita.

**What are the implications of this study for disease understanding and/or clinical care?**

- Laminin β4 has been identified as a new target antigen in anti‐p200 pemphigoid. The development of a sensitive serological assay to detect anti‐laminin β4 antibodies allows the diagnosis of anti‐p200 pemphigoid to be made in many patients whose diagnosis could not be determined using the serological methods previously available.

## INTRODUCTION

The diagnosis of autoimmune bullous diseases (AIBD) of the dermal part of the dermo‐epidermal junction (DEJ) is often challenging. These AIBDs include: (i) epidermolysis bullosa acquisita (EBA) in which autoantibodies are directed against collagen VII, (ii) a subgroup of mucous membranous pemphigoid (MMP) whose target antigen corresponds to laminin 332 and (iii) anti‐p200 pemphigoid.^1^, ^2^, ^3^, ^4^ Anti‐p200 pemphigoid was described in 1997 and is characterized by antibodies (Abs) directed against a 200‐kDa protein by immunoblot on dermal extract.^3^, ^4^ Laminin γ1, which is localized within the lower part of the lamina lucida, has been initially described as the target antigen of anti‐p200 pemphigoid.^5^

As the clinical presentation of patients with anti‐p200 pemphigoid and their direct immunofluorescence (DIF) pattern may be similar to that of patients with bullous pemphigoid (BP), the diagnosis of anti‐p200 pemphigoid mainly relies on the identification of circulating anti‐p200 Abs.^6^, ^7^, ^8^

Because not all patients' sera recognize laminin γ1, it was suspected that another 200‐kDa protein may also be involved.^9^, ^10^ Recently, using liquid chromatography tandem mass spectrometry, the laminin β4 chain was identified as a potential target molecule that was then confirmed using recombinant laminin β4.^6^ Interestingly, most sera from patients with anti‐p200 pemphigoid reacted with laminin β4, regardless of their recognition of laminin γ1, which suggested that laminin β4 might be a more suitable antigen than laminin γ1 for the diagnosis of anti‐p200 pemphigoid. Then, an indirect immunofluorescence (IIF) assay on transfected cells (cell‐based immunoassay, anti‐LAMB4 CBIA) has been developed to detect anti‐laminin β4 Abs.

Until now, only one study has evaluated the incidence of anti‐p200 pemphigoid in Northern Germany, estimating it at 0.7 new cases/million inhabitants/year, which would correspond to a higher incidence than EBA.^11^, ^12^, ^13^

Thus, we aimed in the present study to assess the sensitivity and specificity of this new assay for the diagnosis of anti‐p200 pemphigoid and estimate its incidence relative to that of BP and pemphigus in France.

## MATERIALS AND METHODS

### Selection of sera and patients

All sera received in the immunology laboratory of the French reference centre for AIBD from January 2018 to March 2023, which labelled the dermal side of salt‐split skin by IIF, were included.

To assess the specificity of anti‐LAMB4 CBIA, we also tested 116 sera from patients with various autoimmune diseases: 10 EBA sera containing anti‐collagen VII Abs, 6 MMP sera with anti‐laminin 332 Abs, 50 BP sera, 15 pemphigus sera, 10 systemic lupus sera containing high levels of anti‐DNA Abs, 15 rheumatoid arthritis sera with anti‐CCP Abs and 5 primary biliary cholangitis sera with anti‐mitochondria 2 Abs.

### Immunoblot analysis on dermal extract

Dermal extracts were prepared using human skin samples from mammoplasty incubated overnight in NaCl 1 M and protease inhibitors (Sigma‐Aldrich, St. Louis, MO, USA) to induce detachment between the epidermis and dermis. After detachment, the dermis was lysed with a tampon of urea 8 M, SDS 2%, DTT 0.1 M, TRIS–HCl 0.1 M pH 6.8 and sonicated. Dermal extract supernatant was run on a NuPAGE™ 3%–8% Tris‐Acetate Gel (NuPAGETM, Invitrogen, Waltham, MA, USA) by sodium dodecyl sulphate‐polyacrylamide gel electrophoresis and was then blotted on nitrocellulose membranes (iBlot®, Invitrogen). Immune reactions were carried out with a 1:22 dilution of serum samples in Odyssey blocking buffer (LI‐COR, Bad Homburg, Germany), followed by detection of biotin‐labelled anti‐human IgG‐Fc antibodies (1:2000, Southern Biotech, Birmingham, AL, USA) and streptavidin‐IRDye 800 complex (1:10000, Rockland, Limerick, PA, USA). Immunoblot revelation was performed on Odyssey CLx (LI‐COR).

### Indirect immunofluorescence (IIF) microscopy

IIF on salt‐split skin was performed using normal human skin samples from mammoplasty. Briefly, patients' sera were diluted at 1:20 in PBS and incubated with a mix of FITC‐labelled polyclonal human IgG (Inova Diagnostics, San Diego, CA, USA) and FITC‐labelled monoclonal antibody against human IgG4 (SouthernBiotech, Birmingham, AL, USA).

IIF on anti‐LAMB4 CBIA (Euroimmun®, Lübeck, Germany) were performed following manufacturer's instructions. Briefly, patients' sera were diluted at 1:10 in PBS‐Tween, followed by an incubation with FITC‐labelled antibody against human IgG and IgG4. Slides were examined using a ZEISS AxioPlan 2 fluorescence microscope.

### Enzyme‐linked immunosorbent assays (ELISAs)

Serum levels of IgG Abs against BP180 NC16A, BP230, DSG1, DSG3 and Collagen type VII NC1 were measured using commercial ELISAs (Euroimmun®, Lübeck, Germany), following the manufacturer's instructions.

### Estimation of the incidence of anti‐p200 pemphigoid

The incidence of anti‐p200 pemphigoid was calculated as the product of the annualized incidence rate of BP by the incidence rate ratio (IRR) of anti‐p200 compared to BP. The rationale of this methodological choice was the good validity of the BP and pemphigus incidence data in the literature and the good validity of the incidence ratio in our study, but the high difficulty of obtaining a reliable estimate of the general population size suffering from AIBD related to our study sample, that is the denominator of the incidence rate was unknown. As a sensitivity analysis, the pemphigus was used as reference disease instead of BP.

The immunology laboratory of the French reference centre for AIBD analyses around 5000 sera per year (5278 sera in 2023), which provide from 61 dermatology departments. To assess the incidence of anti‐p200 pemphigoid, relative to that of pemphigus and BP, we selected 12 centres which exhaustively send to our laboratory the sera from all patients with suspected AIBD who are referred to these centres, therefore, guaranteeing that the ratios between AIBDs (e.g. anti‐p200 pemphigoid/BP) is representative of the IRR of these diseases.

We thus recorded the number of newly diagnosed cases of anti‐p200 pemphigoid using the combination of anti‐LAMB4 CBIA test and immunoblotting, and the number of newly diagnosed cases of pemphigus and BP using anti‐DSG1/anti‐DSG3 and anti‐BP180/anti‐BP230 ELISA assays, respectively, in the 12 centres during the study period.

### Statistical analyses

Since we evidenced that the sensitivity of the anti‐LAMB4 CBIA test for the detection of anti‐laminin β4 Abs was very high and close to that of the anti‐DSG1‐3 and anti‐BP180 in pemphigus and BP, respectively, we estimated the IRR of anti‐p200 pemphigoid compared to BP (main analysis) and to pemphigus (robustness analysis) as the number of anti‐p200 pemphigoid cases observed in the samples sent to our referral centres divided by the number of cases observed for BP (main analysis) or the number of cases observed for pemphigus (robustness analysis). Then, we multiplied these IRR by incidence rates of pemphigus and BP that we previously reported.^7^, ^8^ Confidence intervals of the incidence rates of the anti‐p200 pemphigoid were computed by Wald's normal approximation method on the logarithm of the incidence, with the variance calculated by adding the variance of the logarithm of the incidence rate of the literature (for BP or pemphigus) to the variance of the logarithm of the odds of anti‐p200 divided by BP or pemphigus, assuming that the literature sample is independent of our study sample. The robustness analysis was considered successful if the confidence intervals of the two estimators (based on BP and based on pemphigus) of the anti‐p200 pemphigoid incidence rates were overlapping to a large degree.

Sensitivity between assays was compared by a McNemar chi‐square for paired percentages. Analyses were done with R (version 4.2).

### Ethics

The study was performed according to the guidelines of the French Bioethics Law for retrospective non‐interventional research studies after the absence of objection.

## RESULTS

### Specificity of anti‐LAMB4 CBIA

**FIGURE 1:**
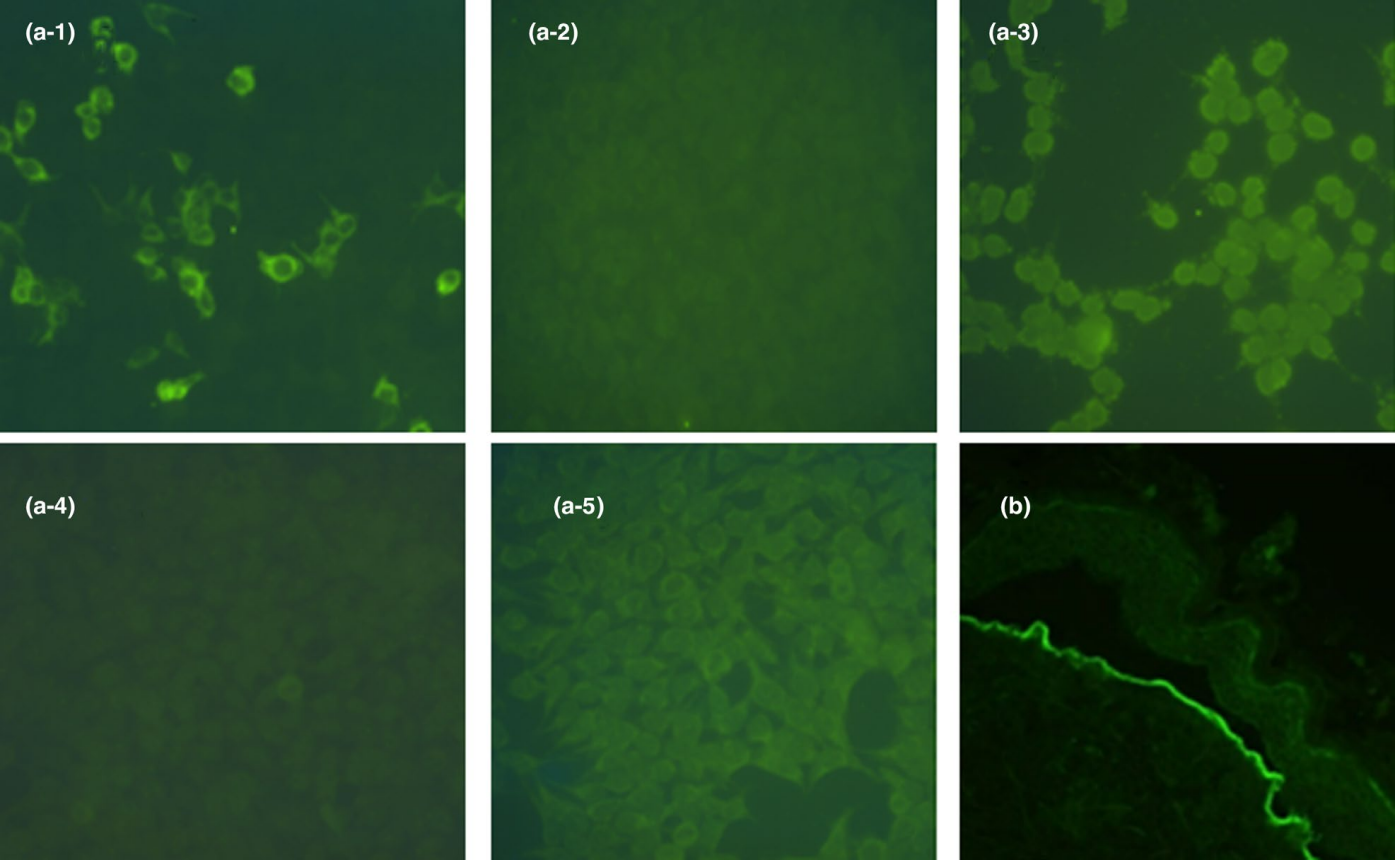
Human salt‐split skin IIF and anti‐LAMB4 CBIA images. (a) Anti‐LAMB4 CBIA images from sera with (1) anti‐LAMB4 aAbs, (2) anti‐BP180 and anti‐BP230 aAbs, (3) anti‐DNA aAbs, (4) anti‐CCP and rheumatoid factor, (5) anti‐mitochondria 2. (b) Anti‐BMZ labelling the dermal side by IIF on human salt‐split skin. aAb, Autoantibody; BMZ, Basement membrane zone; CBIA, Cell‐based immunoassay; IIF, Indirect immunofluorescence; LAMB4, Laminine b4.

To assess the specificity of the anti‐LAMB4 CBIA test, 66 sera from patients with EBA (*n* = 10), MMP (*n* = 6), pemphigus (*n* = 15), systemic lupus (n = 10), rheumatoid arthritis (n = 15) and primary biliary cholangitis (*n* = 5); and 50 sera from BP patients were tested with the anti‐LAMB4 CBIA. Only one BP serum was positive, corresponding to a specificity of 99.1%. Moreover, we did not evidence any issue in interpreting the fluorescence pattern of sera tested with the anti‐LAMB4 CBIA, despite the background staining provided by sera containing anti‐nuclear Abs, Rheumatoid Factor and Abs directed against ubiquitous cytoplasmic antigens as anti‐mitochondria Abs (Figure 1a).

### Sensitivity of anti‐LAMB4 CBIA

**FIGURE 2:**
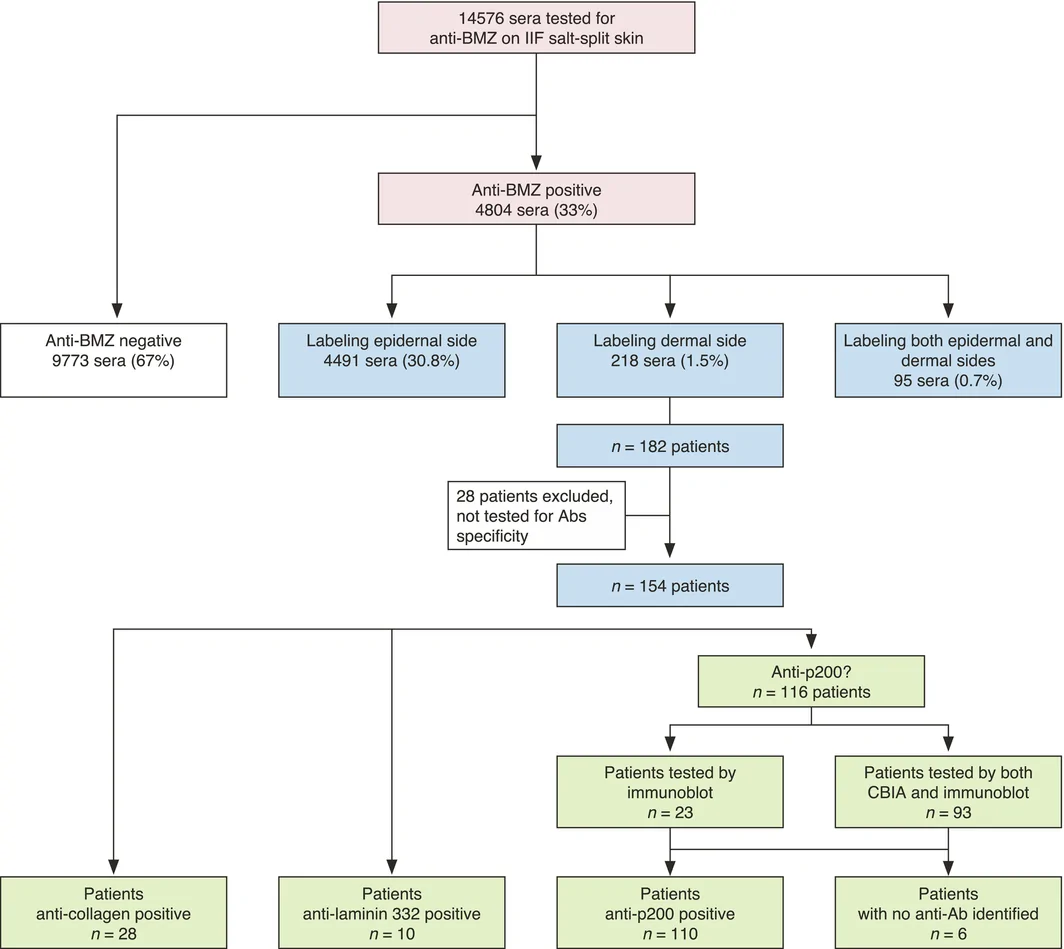
Flow chart for the recruitment of sera and patients with anti‐BMZ labelling the dermal side of human split skin by IIF. aAb, Autoantibody; BMZ, Basement membrane zone, IIF, Indirect immunofluorescence.

**FIGURE 3:**
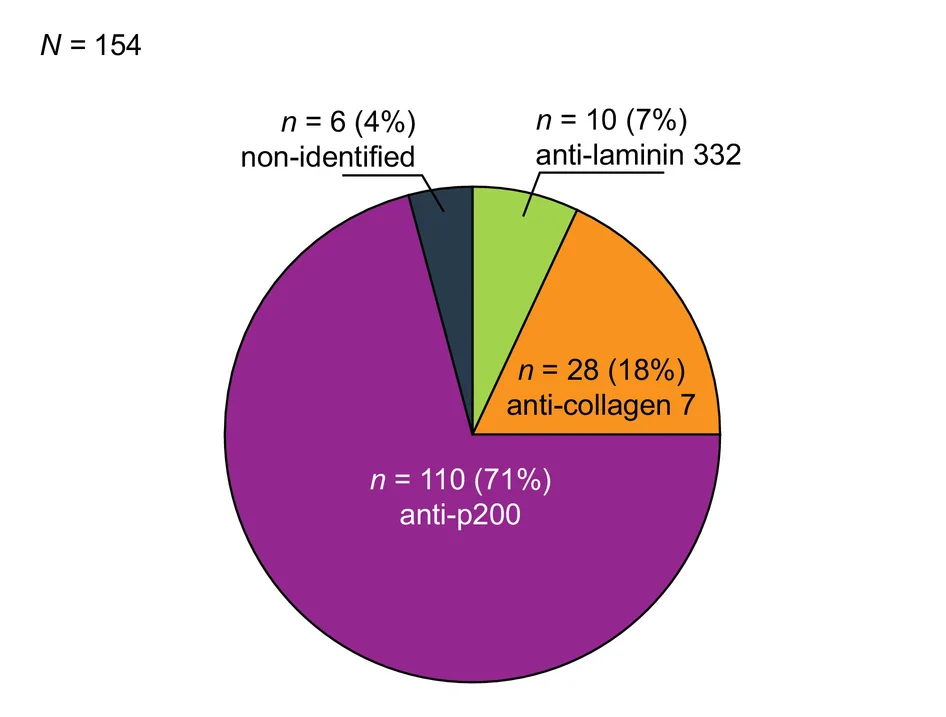
Autoantibody identification of the 154 patients with anti‐BMZ labelling the dermal side of salt‐split skin by IIF recruited during the 63 month‐period. BMZ, Basement membrane zone, IIF, Indirect immunofluorescence.

Among the 14,576 sera analysed for anti‐BMZ Abs in our laboratory from January 2018 to March 2023, 218 sera labelling the dermal side of salt‐split skin by IIF were selected for the study, corresponding to 182 patients (Figures 1b and 2). After excluding patients with unavailable sera (*n* = 28), patients with sera containing anti‐laminin 332 Abs (*n* = 10) or anti‐collagen type VII Abs (n = 28), 116 patients' sera potentially contained anti‐p200 Abs. Among them, 93 patients' sera were studied by both immunoblotting on human dermal extracts and anti‐LAMB4 CBIA while sera from 23 other patients, already known to be positive by immunoblotting, were not assessed by anti‐LAMB4 CBIA test due to insufficient serum volume (Figure 2). Out of the 93 patients' sera assessed by the combination of CBIA and immunoblotting, anti‐p200 Abs were detected in 85 patients (91%) with CBIA (corresponding to anti‐laminin β4 Abs) versus 44 patients by immunoblotting (47%, *p* < 0.0001) (Table 1). Notably, anti‐laminin β4 Abs were detected in 43 patients which were negative by immunoblot, while only two patients' sera recognized a 200 kDa band in immunoblot on dermal extracts, while being negative when tested with the anti‐LAMB4 CBIA test. Overall, using the combination of immunoblotting + anti‐LAMB4 CBIA test, anti‐p200 Abs were detected in 110/116 (94.8%) patients with anti‐BMZ labelling the dermal side of salt‐split skin by IIF and without circulating anti‐collagen type VII nor anti‐laminin 332 Abs. Six patient’ sera (6.5%) remained negative in both assays (Table I). When considering the whole number of patients with anti‐BMZ labelling the dermal side of salt‐split skin, anti‐p200 Abs were 4‐ and 10‐fold more frequently detected, than anti‐collagen type VII and anti‐laminin 332 Abs, respectively (Figure 3).

**TABLE 1:** Evaluation of sensitivities of immunoblot with dermal extract and anti‐LAMB4 CBIA for anti‐p200 detection in patients with anti‐BMZ labelling the floor of split skin (*n* = 93).

		Anti‐LAMB4 CBIA		
		Positive	Negative	
Immunoblot	Positive	42	2	44
	Negative	43	6	49
		85	8	93

Abbreviations: BMZ, Basement membrane zone; CBIA, Cell‐based immunoassay; LAMB4, Laminine b4.

### Estimation of the incidence of anti‐p200 pemphigoid

To estimate the incidence of anti‐p200 pemphigoid, we selected 12 centres which exhaustively sent all sera from patients referred for AIBD to be analysed in our laboratory, therefore, including sera from patients with BP and pemphigus. Considering the exhaustivity of the serological recruitment from these centres, and the reported incidence rates of BP and pemphigus in France, we used this collection to estimate the incidence of anti‐p200 pemphigoid relative to BP and pemphigus. Seventy‐three patients from these centres have sera that labelled the dermal side of salt‐split skin by IIF and 51 contained anti‐p200 Abs detected either with the anti‐LAMB4 CBIA test or by immunoblotting. During the same period, a serological diagnosis of BP or pemphigus was made in 1777 and 291 cases from these 12 centres, respectively, leading to incidence rate ratios of 51/1777 = 0.0287 and 51/291 = 0.175 compared to BP and pemphigus, respectively. Given the incidence rates of BP and pemphigus in France (21.7 (95% CI: 19.8–23.7) and 1.85 (95% CI: 1.63–2.08) new cases/million inhabitants/year), the incidence rate of anti‐p200 pemphigoid was estimated at 0.62 (95% CI: 0.46–0.83) new cases/million inhabitants/year based on BP (main analysis) and 0.32 (95% CI: 0.23–0.45) new cases/million inhabitants/year based on pemphigus (robustness analysis). Since the confidence intervals did not overlap, the consistency between the main analysis and robustness analysis was considered poor. Assuming the truth is between extrema of these confidence intervals, the incidence rate of anti‐p200 pemphigoid was assumed to be between 0.23 and 0.83 new cases/millions inhabitants/year and 16–57 new cases/year in the 68.4 million inhabitants in France.

## DISCUSSION

This study confirmed the value of the newly developed LAMB4 CBIA test for the diagnosis of anti‐p200 pemphigoid and suggested that its frequency is much higher than initially expected.

First, we found a very high specificity of 99.1%, which was very close to the 99.3% reported by Goletz et al.^12^ Even though we voluntarily tested sera containing autoantibodies that could generate a background of fluorescence staining such as anti‐mitochondrial Abs, anti‐DNA Abs or rheumatoid factor, IIF images on anti‐LAMB4 CBIA were easy to read, allowing us to confirm the high specificity of this new assay.

By selecting sera which labelled the dermal side of salt‐split skin and did not contain anti‐laminin 332 nor anti‐collagen type VII Abs, we showed the high sensitivity of CBIA (91%), which was in fact much higher than that of immunoblot analysis of the corresponding sera on dermal extracts (47%). This much higher sensitivity of LAMB4 CBIA test over immunoblotting may be partly related to the variability in laminin β4 expression depending on skin location,^8^ while we exclusively used skin samples from mammoplasty to prepare the dermal extracts in our immunoblotting experiments. Also, immunoblotting, which used denaturized extracts, does not allow detection of Abs directed against conformational epitopes. Finally, the lower sensitivity of immunoblotting related to LAMB4 CBIA test is supported by the observation that most immunoblot negative sera only exhibited a weak fluorescence pattern with the anti‐LAMB4 CBIA test. Conversely, sera with a 200 kDa band in immunoblot gave a strong fluorescence pattern using the anti‐LAMB4 CBIA test (Figure S1).

Finally, the combination of immunoblot and the anti‐LAMB4 CBIA test only slightly increased the sensitivity of the detection of anti‐p200 Abs relative to the anti‐LAMB4 CBIA test alone, since only 2 sera were positive in immunoblotting while negative with the LAMB4 CBIA test, arguing for using this latter assay as first line in clinical practice.

We acknowledge that the inclusion criterion based on the positivity of dermal side by IIF on salt‐split skin in this serologic retrospective study might underestimate the true incidence of anti‐p200 pemphigoid.

First, we deliberately excluded patients whose anti‐BMZ antibodies labelled both the dermal and epidermal sides of salt‐split skin (Figure 2). The primary objective of this study was to include only patients whose serological profile allowed for the reliable identification of anti‐p200 pemphigoid. However, based on serological data alone, it is not possible to precisely determine the type of AIBD affecting patients with double labelling, who may potentially have anti‐BP180, anti‐BP230, anti‐α6β4 integrin and/or other unidentified antibodies.

We analysed a subset of the population with double staining and found that 82% of them had anti‐p200 antibodies by immunoblot and/or CBIA, in association with at least another autoantibody targeting a protein of the epidermal side (Figure S2). However, the question of whether these patients have (i) anti‐p200 pemphigoid and subsequently develop other antibodies through epitope spreading or (ii) BP who secondarily develop anti‐p200 or (iii) another form of AIBD remains unresolved.^13^ For this reason, we chose not to consider these patients in our study.

Extrapolating to our initial total population, we estimate that approximately 73 additional patients may have anti‐p200 antibodies which would be added to the 110 included in the study, representing a potential underestimation of the incidence by around 40%. Thus, although our results demonstrate a higher incidence than previously assumed, they likely represent the lower margin of the incidence of P200 pemphigoid, as they do not include patients whose sera exhibit both dermal and epidermal labelling.

Second, given the high sensitivity of the LAMB4 CBIA test, it might be that a few patients among those with sera negative by IIF on salt‐split skin (Figure 2) might be positive for anti‐laminin β4 using the LAMB4 CBIA.^12^, ^13^

All these findings led us to reconsider the frequency of anti‐p200 pemphigoid relative to other AIBDs of the dermal side of the DEJ. Indeed, from our serum samples, anti‐p200 Abs were 4‐ and 10‐fold more frequently detected than anti‐collagen VII and anti‐laminin 332 Abs, respectively. Although the sensitivity of anti‐collagen VII ELISA is low (30%–54%),^7^, ^8^ for the diagnosis of EBA, these findings are in agreement with previous results indicating that anti‐p200 pemphigoid is the most common pemphigoid disease in patients with anti‐BMZ labelling the dermal side of split skin by IIF.^14^

These results are in accordance with our estimation of the incidence rate of anti‐p200 pemphigoid that we calculated as probably between 0.23 and 0.83 new cases/million inhabitants/year, which is very close to the incidence rate of 0.7 new cases/million inhabitants/year reported in the Northern regions of Germany (Schleswig‐Holstein). They are also in accordance with the incidence of EBA which is estimated at between 0.17 and 0.26 new cases per million inhabitants per year,^11^, ^12^ suggesting that the anti‐p200 pemphigoid is 2‐ to 4‐fold more frequent than EBA. Even if we considered the hypothesis that the 6 sera which did not contain anti‐p200 pemphigoid Abs, nor anti‐collagen, nor anti‐laminin 332 Abs would in fact correspond to EBA sera, this would not change much the greater incidence of anti‐p200 pemphigoid over EBA. Moreover, we would like to emphasize that our study does not include patients whose sera contain anti‐P200 antibodies but show dual labelling of the dermal and epidermal sides, for whom it is impossible to determine the specific type of subepidermal AIBD. A more accurate estimation of the incidence of anti‐p200 pemphigoid in France would require a study based on direct incidence calculation using clinical and biological data.

Overall, this study showed that anti‐p200 pemphigoid is much more frequent than initially estimated. The easy diagnosis of this disease with the LAMB4 CBIA test should allow carrying out larger homogeneous series of patients in order to evaluate the effect of therapeutics more accurately.

## AUTHOR CONTRIBUTIONS

FJ conceived the study and wrote the manuscript. VH and PJ supervised the study and revised the manuscript. MC performed incidence analysis and revised the manuscript. MM, MLG and AL revised the manuscript.

## FUNDING INFORMATION

This work was supported by Rouen University Hospital.

## CONFLICT OF INTEREST STATEMENT

P. Joly is consultant for Amgen, Argenx, Astra Zeneca, Thermofisher, Sanofi, Akari, Janssen, Novartis, Servier, Chugai, Kezar Life Science, Regeneron, UCB, Principabio, Zenyaku Kogyo, Dualyx, Amgen, JJP Biologics. V. Hébert is consultant for Astra Zeneca, Pfizer, Almirall, UCB, Lilly, AbbVie, BMS. FJ, MC, MLG, MMV, AL, AG declare no conflict of interest.

## ETHICAL APPROVAL

This study was approved by the local ethics committee ‘CPP Nord‐Ouest’.

## ETHICS STATEMENT

The study was performed according to the guidelines of the French Bioethics Law for retrospective non‐interventional research studies after the absence of objection.

## Supporting information


Data S1.

Data S2.

Data S3.

## Data Availability

Data supporting the findings of this study are available on request from the corresponding author. The data are not publicly available due to privacy or ethical restrictions.
