# Supplementary material for: Incidence of P200 pemphigoid: A nationwide study
Source: J Eur Acad Dermatol Venereol. 2026 Mar 7;40(10):1675–81. doi: 10.1111/jdv.70370 (PMC13613351; doi:10.1111/jdv.70370)
Supplement: Supplementary file 1 [file JDV-40-1675-s001.docx]

Supplemental data 3

|  | |  | anti-LAMB4 CBIA | |  |
| --- | --- | --- | --- | --- | --- |
|  | |  | Positive | Negative |  |
| Immunoblot | Positive | | 43 | 2 | 45 |
|  | Negative | | 50 | 7 | 57 |
|  | |  | 93 | 9 | 102 |

**Table S1**: Evaluation of sensitivities of immunoblot using dermal extract and anti-LAMB4 CBIA for anti-p200 antibody detection in patients with anti-BMZ antibodies labeling the dermal side of salt-split skin (n=102).

LAMB4: laminine b4, CBIA: cell-based immunoassay, BMZ: basement membrane zone.
