# Supplementary material for: Incidence of P200 pemphigoid: A nationwide study
Source: J Eur Acad Dermatol Venereol. 2026 Mar 7;40(10):1675–81. doi: 10.1111/jdv.70370 (PMC13613351; doi:10.1111/jdv.70370)
Supplement: Supplementary file 2 [file JDV-40-1675-s002.docx]

Supplemental data 2

**Figure S2.**

Flow chart assessing the frequency of patients' sera positive for anti-p200 among sera with anti-BMZ labelling both dermal and epidermal side of human salt-split skin by IIF.

BMZ: basement membrane zone, aAb: autoantibody, IIF: indirect immunofluorescence.
